# Multimodality Functional Imaging in Radiation Therapy Planning: Relationships between Dynamic Contrast-Enhanced MRI, Diffusion-Weighted MRI, and 18F-FDG PET

**DOI:** 10.1155/2015/103843

**Published:** 2015-02-19

**Authors:** Moisés Mera Iglesias, David Aramburu Núñez, José Luis del Olmo Claudio, Antonio López Medina, Iago Landesa-Vázquez, Francisco Salvador Gómez, Brandon Driscoll, Catherine Coolens, José L. Alba Castro, Victor Muñoz

**Affiliations:** ^1^Medical Physics Department and Radiological Protection, Galaria-Hospital do Meixoeiro-Complexo Hospitalario Universitario de Vigo, 36200 Vigo, Spain; ^2^Signal Theory and Communications Department, University of Vigo, 36310 Vigo, Spain; ^3^Radiation Medicine Program, Princess Margaret Cancer Centre and University Health Network, Toronto, ON, Canada M5T 2M9; ^4^Radiation Oncology Department, Galaria-Hospital do Meixoeiro-Complexo Hospitalario Universitario de Vigo, 36200 Vigo, Spain

## Abstract

*Objectives*. Biologically guided radiotherapy needs an understanding of how different functional imaging techniques interact and link together. We analyse three functional imaging techniques that can be useful tools for achieving this objective. *Materials and Methods*. The three different imaging modalities from one selected patient are ADC maps, DCE-MRI, and 18F-FDG PET/CT, because they are widely used and give a great amount of complementary information. We show the relationship between these three datasets and evaluate them as markers for tumour response or hypoxia marker. Thus, vascularization measured using DCE-MRI parameters can determine tumour hypoxia, and ADC maps can be used for evaluating tumour response. *Results*. ADC and DCE-MRI include information from 18F-FDG, as glucose metabolism is associated with hypoxia and tumour cell density, although 18F-FDG includes more information about the malignancy of the tumour. The main disadvantage of ADC maps is the distortion, and we used only low distorted regions, and extracellular volume calculated from DCE-MRI can be considered equivalent to ADC in well-vascularized areas. *Conclusion*. A dataset for achieving the biologically guided radiotherapy must include a tumour density study and a hypoxia marker. This information can be achieved using only MRI data or only PET/CT studies or mixing both datasets.

## 1. Introduction

Radiotherapy is in a process of transformation from image-guided radiotherapy to biologically guided radiotherapy [1]. To this effect, in the last few years some commercial treatment units have been developed that include an MRI unit combined with a linac in a single device [2–5], and PET/CT (positron emission tomography/computed tomography) has proven useful for tumour staging and target delineation, especially in head and neck tumours and lung tumour [6–8]. The main change in clinical practice will arrive when the prescription of a treatment moves from dose prescribed to target volumes and to prescription of results of a function, as tumour control probability, which considers all the radiobiological phenomena and adapts the treatment to early tumour response and uses different functional images as inputs. Although not widely available yet, several tools, methods, and models have been developed for achieving these objectives in a retrospective manner:

1. quantitative methods in diffusion-weighted imaging- (DW-) MRI providing ADC (apparent diffusion coefficient) maps that allow determining early tumour response [9–13],
2. in vivo measurement of hypoxia [14, 15], using either MRI datasets [16–22] or PET/CT [23–28],
3. inverse-planning optimisation algorithm that includes biological criteria [29, 30] and/or functional imaging information [31, 32] or even radiobiological models adapted to functional imaging information [33].

In this paper a case study is presented using datasets from 18F-FDG (fludeoxyglucose labelled with 18F) PET/CT, DW-MRI/ADC maps, and dynamic contrast-enhanced- (DCE-) MRI for characterizing tumour behaviour and for using the multimodality parameters as predictive values of tumour response from a patient included in the ARTFIBio project [34–36].

18F-FDG PET images the glucose consumption of each region. Tumour cells use glycolysis rather than lipolysis as the metabolic process to produce ATP and they use more glucose than normal cells. Glycolysis is a rather inefficient process and therefore large amounts of glucose are needed for cell survival and tumour growth. The PET enhancement (standard uptake value or SUV) in tumours is due to three different mechanisms: (i) cancer cells produce more ATP outside the mitochondria, even in well-oxygenated conditions (Warburg effect [37]); (ii) cancer cells proliferate more than normal tissue cells [38], and then they need more glucose; and, finally, (iii) cancer cells can survive in lower oxygenated regions better than normal tissue cells [39, 40] but consume more glucose because they need to produce ATP by glycolysis in absence of oxygen (Pasteur effect).

DW-MRI measures the diffusion of protons in a medium. Its principle is based on the attenuation of the signal according to Stejskal and Tanner's model [41]. Tumour cells are abnormal in size and shape compared to normal cells, and they are more tightly packed and have higher cellularity than the tissue from which they originate. The extracellular volume is smaller in tumour regions, and therefore the freedom of movements of protons in tumour regions is restricted [42, 43]. The logarithm of the signal attenuation is a function of the applied gradient, the gap between pulses of gradient, and the pulse duration. By varying these parameters during acquisition, the ADC can be calculated for each voxel.

DCE-MRI has been proposed by several authors for treatment monitoring [44–46] and for measurement of oxygenation distribution [19–22]. The main problem is the complexity of the data analysis and the correspondence between measurement and biological parameters. Another disadvantage is the necessity of a contrast agent.

In order to characterize the tumour and to implement new predictive models based on functional imaging data, we must ensure we can extract as much information as possible from the available data. Some of the main parameters to characterize tumour behaviour, along with radiotherapy treatment, must be initial tumour density, hypoxia, malignancy/proliferation, dose to each voxel, and timing of the dose. In this work, attention is focused on showing the relationship between ADC maps, DCE-MRI parameters, dose, and 18-F-FDG PET/CT SUV (standard uptake value). Many other types of images can show the main parameters we are interested in modelling (18F-fluorothymidine for proliferation [47], Zr-89-cetuximab for response to chemotherapy [31], and dynamic FDG [28] and fluoromisonidazole (FMISO) [26] for hypoxia), but it is hypothesized that the proposed combination of techniques can give us enough information about the tumour environment to assess the treatment response, but not the tumour microenvironment (data are averaged into the voxel size): the *k*_trans⁡_ parameter in DCE-MRI is related to vascularization and then to hypoxia [18] and *v*_*e*_ is related to extracellular volume and in heterogeneously vascularized areas to tumour density [18]; SUV is related to tumour metabolism and then is related to malignancy (enhancement of the Warburg effect to the Pasteur effect), hypoxia (Pasteur effect), and tumour density and proliferation. Finally, ADC maps are related to water mobility and then to tumour density [12]. We will explore the relationships between ADC, DCE-MRI parameters, and SUV values and will evaluate their influence on tumour response in a case study where we have in the same slice a necrotic volume, a hypoxic area, and a heterogeneously vascularized tumour volume.

## 2. Material and Methods

### 2.1. Patients

This study is conducted in accordance with the Declaration of Helsinki [48] and the study protocol was approved by the local ethics committee; informed consent was obtained from all patients.

**Figure 1:**
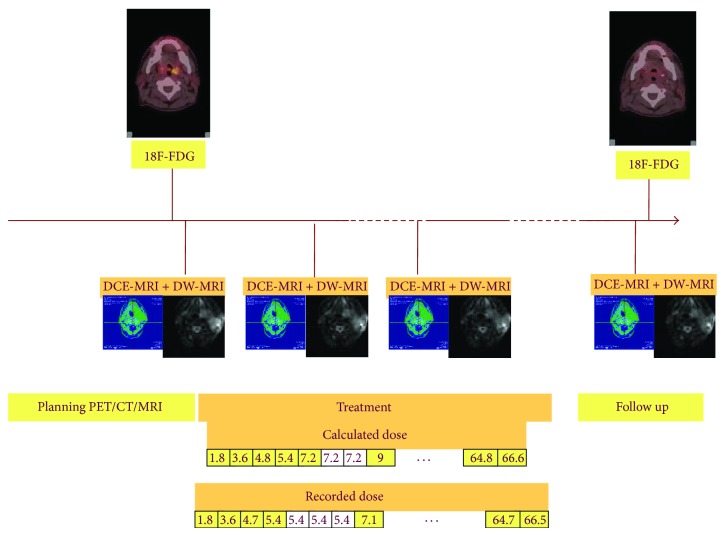
Scheme of the image acquisition process along the radiotherapy course.

The aim of ARTFIBio project (http://artfibio.cesga.es/Artfibio/application/) is to create a network for sharing information and for developing predictive individualized models of the tumour response to radiotherapy in patients with head and neck cancer based on in vivo functional data. For this purpose, several studies of MRI and PET/CT were performed. Patients within the ARTFIBio project [34–36] had oropharyngeal cancer (squamous cancer cell) of stages T3 and T4. All of them are treated with IMRT (intensity-modulated radiation therapy) and the prescribed dose was between 66 Gy and 70 Gy to the local PTV. The imaging protocol (Figure 1) is as follows:

- pretreatment: MRI study (DCE-MRI + ADC) and PET/CT study (18F-FDG),
- first control (10–30 Gy): MRI study (DCE-MRI + ADC),
- second control (30 Gy–60 Gy): MRI study (DCE-MRI + ADC),
- three months after the treatment: PET/CT and MRI study (DCE-MRI + ADC).

For all imaging studies the patient is positioned using the RT immobilisation devices. The geometrical distortion on MRI images and registration process (rigid registration and deformable registration) were checked with an MRI phantom. Regardless, only central slices showing low distortion were analysed. For each patient and each set of images the ADC values, contrast exchange coefficients (*K*_trans⁡_), SUV, dose, and Hounsfield units (HU) per voxel were recorded of each volume.

In this paper a case study is highlighted from one patient who has three clearly differentiated volumes in a single slice: a heterogeneously vascularized tumour and a hypoxic region surrounding a necrotic area. This case is very useful to visualize and investigate the different behaviours of the tumour volumes in glucose metabolism and in treatment response.

### 2.2. Acquisition and Analysis of MR Images

All MRI examinations were performed on a 1.5-T scanner (Achieva; Philips Healthcare) with the patients in supine position. Routine T2-weighted, T1, DW-MRI, and DCE-MRI were obtained using the parameters showed in Table 1. Flex-L coil (Philips Sense Flex Medium) was placed over the neck. After image acquisition, pixel-to-pixel ADC map was reconstructed using the standard software on the imaging console (Achieva; Philips Healthcare). According to Stejskal and Tanner's model [41] and considering the monoexponential approximation, the ADC value can be calculated using the following:

(1) $$\begin{array}{c} \text{ADC}=\frac{\ln\left(S_{0}/S_{1}\right)}{\left(b_{1}-b_{0}\right)}, \end{array}$$

where *S*_1_ and *S*_0_ are signal values of the images at *b* values, *b*_1_ and *b*_0_, respectively, and ADC is the apparent diffusion coefficient obtained using *b*_1_ = 600 and *b*_0_ = 0.

A nonlinear model [49] was utilized to convert signal to gadolinium concentration in DCE-MRI as per Tofts [50]. It considers two different compartments: the blood plasma (or intravascular space) and the extracellular extravascular space (EES or interstitial space). The parameters utilized to generate the Tofts model are described in Table 2.

The relationship between all these parameters can be obtained by

(2) $$\begin{array}{c} C_{t}\left(t\right)=\frac{K_{\mathrm{trans}}}{1-H_{ct}}\left(C_{a}\left(t\right)⊗e^{-K_{ep}\left(t-\tau \right)}\right)+V_{b}C_{a}\left(t\right). \end{array}$$

A voxel-based perfusion analysis method was used based on the modified Tofts model [49]. A 3D voxel-wise perfusion analysis method [51, 52] was applied to the DCE-MRI data which generated perfusion parameters *k*_trans⁡_, *k*_*ep*_, and *v*_*b*_ from the modified Tofts model. This method also provided semiquantitative metrics such as area under the curve (AUC) and time to max enhancement.

Variable flip angle (VFA) spoiled gradient recalled echo scans at three flip angles variations (5°, 10°, and 15°) were utilized to calculate the voxel by voxel T1_0_ of the GTV (gross tumour volume) of 3 different patients. The average T1_0_ of these patients (800 ms) was applied when calculating the concentration of the analyzed patient which unfortunately did not have VFA scans themselves.

The arterial input function (AIF) was chosen in the carotid artery near the base of skull.

### 2.3. Acquisition of PET/CT Images

Whole-body PET/CT scan was carried out from head to thigh, 60 min after intravenous administration of approximately 370 MBq (±10%) of 18F-FDG on a PET/CT scanner (Discovery, GE Healthcare Bio-Sciences Corp.) with a 70 cm axial FOV, a 218 × 218 matrix. Study was acquired in 3D mode. The pixel spacing was 5.47 mm with a slice thickness of 3.27 mm. The spatial resolution to 1 cm varies from 3.99 mm to 4.56 mm. PET images were corrected for attenuation, scatter, decay, dead time, random coincidences, and slice sensitivity.

To calculate the SUV [53] for the selected patient and on a voxel by voxel basis, we took into account an injected activity of 345 MBq with a weight of the patient of 49 kg.

### 2.4. Noise Reduction and Registration

To reduce image noise a 3 × 3 nearest-neighbour smoothing filter was applied to the DCE-MRI, PET-CT, and ADC images. Deformable registration of the images, with the CT of treatment as reference, was performed using tailored in-house software specifically developed for the ARTFIBio project [36] and based on ITK libraries [54]. Using the GTV contoured for radiation treatment, the numerical values of each voxel of the coregistered images were extracted. Bone and air voxels (as determined by CT) were dropped from the analysis profiles.

## 3. Results and Discussion

### 3.1. SUV versus ADC

**Figure 2:**
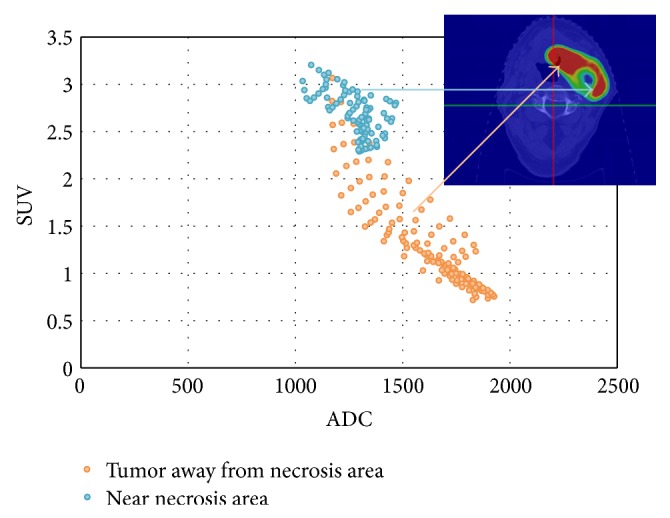
The relationship between SUV and ADC. In the hypoxic area (excluding necrotic area), high SUV values are obtained independently of the ADC value; this is explained by the addition of the Warburg effect and the Pasteur effect. In the heterogeneously vascularized area, SUV values decrease with ADC. This is likely a result of the fact that a reduction in ADC implies an increase in tumour cell density.

The relationship between the different image datasets and functional parameters was investigated in order to achieve the best possible picture of the internal tumour dynamics. Using one representative patient a plot of SUV versus ADC for the CTV is displayed in Figure 2; the hypoxic area (low ADC, low *K*_trans⁡_), surrounding necrotic volume (medium-high ADC, low *K*_trans⁡_), and heterogeneously vascularized tumour (low ADC, high *K*_trans⁡_) have each been considered separately (Figure 2).

### 3.2. ADC versus *v*_*e*_ (DCE-MRI)

Several parameters can be obtained from DCE-MRI, but only the relationships between *K*_trans⁡_ and extracellular volume *v*_*e*_ have been investigated here.

In order to perform kinetic modeling of the tumour robust arterial input function (AIF) needs to be selected.

**Figure 3:**
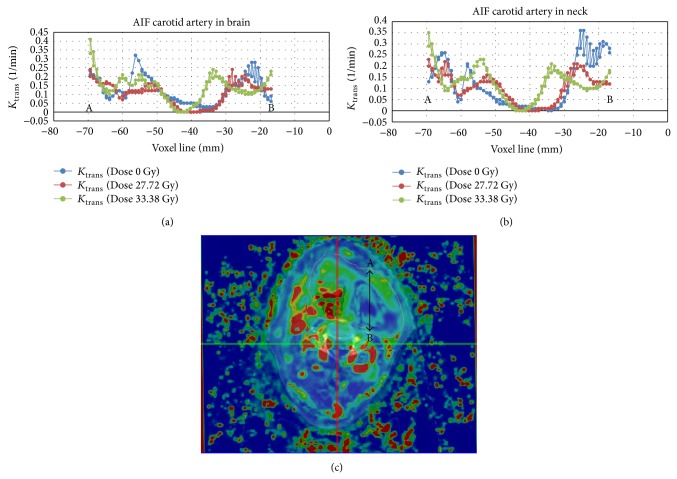
In this axial slice (bottom), *k*_trans⁡_ is represented along (top) a voxel line at different stages of treatment (pretreatment, at 27.72 Gy, and at 33.38 Gy). We can see how *k*_trans⁡_ increases with dose and the central U-shaped valley corresponding to the badly vascularized area is becoming increasingly narrow. In the upper left figure, we consider AIF from data of the carotid artery near brain, and in the lower left figure, we consider AIF from data of the carotid artery in neck.

The AIF was chosen in the carotid artery near the base of skull for increased reproducibility since a larger variability was observed in the values of T1_0_ in the carotid at the level of the neck (Figure 3).

**Figure 4:**
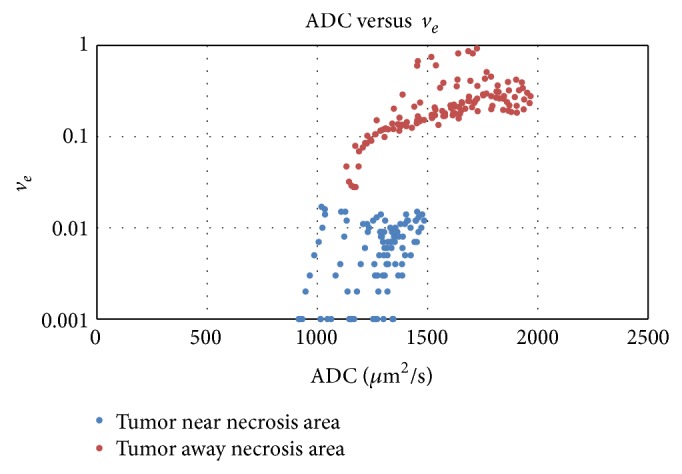
*v*_*e*_ versus ADC (*µ*m^2^/s) for the selected slice of the patient of Figure 3. In well-vascularized areas (red dots), a clear relationship can be found.

*v*_*e*_ should be most closely correlated to ADC information as the extracellular volume is related to the freedom of water molecules in the medium. Both sets of data were compared and they are represented in Figure 4.

For values of *v*_*e*_ greater than 0.02 (values less than this value correspond to badly vascularized areas and low *K*_trans⁡_ in the studied data), a clear relationship between both datasets is found, indicating that a smaller extracellular volume corresponds to a higher tumour cell density in well-vascularized or heterogeneously vascularized areas but not hypoxic areas.

### 3.3. SUV, ADC versus *k*_trans_ (DCE-MRI)

**Figure 5:**
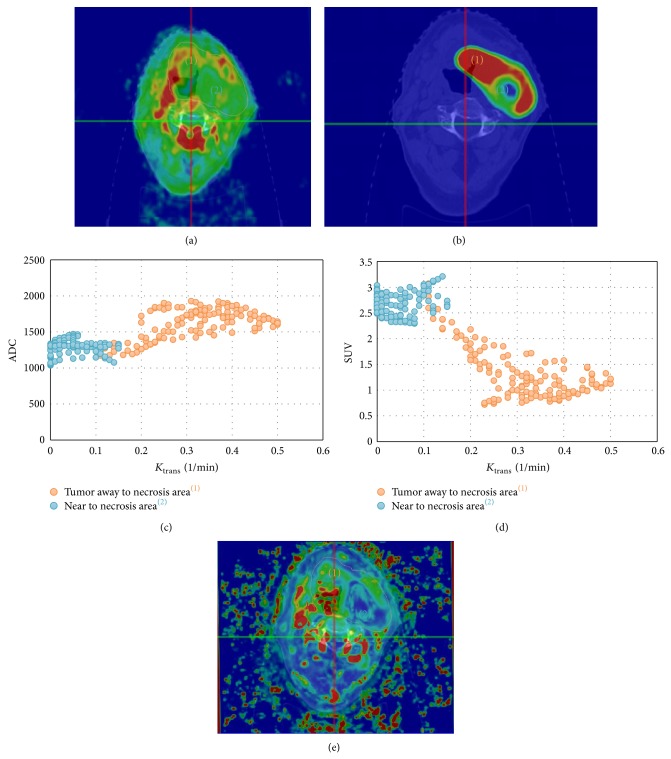
In this figure SUV versus *K*_trans⁡_ and ADC versus *K*_trans⁡_ are represented. (a) *K*_trans⁡_ map overlaid on the simulation CT. (b) PET/CT. (c) In the hypoxic area (near necrotic area), high SUV values are obtained independently for all low *K*_trans⁡_ values, because of the addition of the Warburg effect and the Pasteur effect. In the heterogeneously vascularized area, SUV values are decreasing with *K*_trans⁡_, as expected, because the Pasteur effect is reducing in this area as *K*_trans⁡_ increases. (d) No clear relationship can be found between ADC and *K*_trans⁡_. (e) ADC map overlaid simulation CT.

Of all the analyzed parameters, *k*_trans⁡_ is the most related to vascularization. Vascularization must be related to oxygenation [55–59], as Figure 5 shows, because with increasing *K*_trans⁡_ values, that is, increasing perfusion, SUV values decrease because of the reduction of the Pasteur effect (green dots, Figure 5(c)).

On the other hand, no clear relationship has been found between ADC map and *k*_trans⁡_ values, although ADC values appear to be rather constant (blue dots, Figure 5(d)) because they are selected from a small homogeneous region. Additionally, tumour cells are able to survive in badly oxygenated areas and the tumour cell density is less variable in these areas.

### 3.4. ADC versus Dose Influenced by *K*_trans_

**Figure 6:**
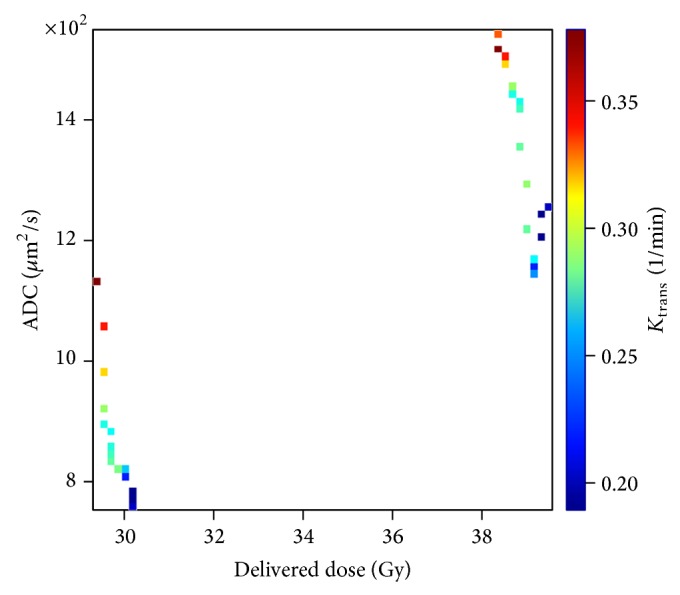
ADC values for a heterogeneously vascularized tumour volume are represented versus delivered dose (fractions 13th and 17th), and the colour represents the *k*_trans⁡_ value. In this graph, it can be observed that heterogeneously vascularized voxels show a greater increment in ADC values.

We have generated the ADC values during the treatment for a heterogeneously vascularized tumour volume. In this case, the delivered dose to achieve an ADC value corresponding to normal tissue is much lower than for badly vascularized voxels. The influence of vascularization/oxygenation in the ADC response can be observed with the DCE-MRI studies, as shown in Figure 6.

### 3.5. Discussion

The results presented have some similarities to those obtained by Atuegwu et al. [12] and indicate that ADC values can be a good marker of the tumour response [11, 12]. Further, the combination of biological information obtained from different modalities can improve the characterization of tumour behaviour. From our point of view, at least two different sets of data must be considered: one for tumour response and another one for hypoxia measurement. If geometrical distortion is not considered or can be corrected [60], ADC maps can be a suitable choice for tumour response.

The polarographic electrode has been considered by some authors as the gold standard for measuring tumour hypoxia in vivo [61], although theoretical simulations have shown that it gives only a qualitative characterisation. Considering only radiopharmaceuticals and PET/CT, the most common are FMISO [24, 26, 32, 33], dynamic FDG [28], and Cu-ATSM [47]. When considering MRI, typically BOLD [17] and DCE-MRI [19, 20, 22, 62] are the most widely used methods; however we have not found any study using them for modifying the treatment (as with FMISO [26, 33, 63]). Vascularity measurements from DCE-MRI data can provide a surrogate marker of tumour hypoxia, as was shown by Newbold et al. [20] and Donaldson et al. [21] in head and neck cancer. These measurements could potentially guide treatment [22] and are easy to obtain; however more studies are needed in order to apply to clinical practice, as input data either for dose painting or for delimiting hypoxic volumes.

18F-FDG shows different aspects of the tumour behaviour, mainly associated with tumour cell density, malignancy, and oxygenation, and the quotient between ADC and SUV has been proposed as a measurement of malignancy in breast tumours [64] and in invasive ductal cancer [65]. These last papers found correlation between maximum SUV and bad prognoses that could be explained because high SUV can be associated with hypoxic areas as we have observed.

Using biomechanical models [66] that consider both the dynamics of the tumour and variation of tumour density (including diffusion) and oxygenation along the treatment, instead of static models, can be quite useful for increasing the predictability of the models.

ADC maps appear to be a good option for evaluating tumour response; however their disadvantage is image distortion. Unfortunately, this cannot be corrected using standard deformable registration algorithm, but reversed gradients method looks like a very promising algorithm to solve this problem [60]. It is possible that extracellular volume calculated from DCE-MRI can be used as an equivalent of ADC values in well-vascularized areas.

## 4. Conclusions

Multimodality imaging offers much more information about tumour behaviour than the individual datasets on their own. The relationship between different types of images must be studied in detail in order to establish a minimum set of data required to personalize the radiotherapy treatment and to optimize the treatment for each patient. This could comprise not only a gradient of dose along the treatment, but also different fractionation for each voxel.

Multicentre studies can be useful for recruitment of a large number of patients and increase the statistical power of the results, if imaging standards and protocol compliance are followed [67].

Voxel by voxel analysis seems possible if we consider small volumes and undistorted regions from ADC maps or corrected data.

## Figures and Tables

**Table 1:** Main parameters of MRI acquisition protocols.

Technique	TR/TE (ms)	Field of view (cm^2^)	Matrix size	Slice thickness (mm)	Gap	Sense factor	Contrast agent
T1-Turbo Spin Echo	425/4.8	23 × 23	272 × 272	6	1	1.6	—
T2-Turbo Spin Echo	6171/90	23 × 23	320 × 312	6	1	1.6	—
ADC *b* = 0, 600 s/mm^2^	5270/77	25 × 25	120 × 97	6	1		—
ADC *b* = 0, 1000 s/mm^2^	5926/85	25 × 25	120 × 97	6	1		—
DCE-MRI-Dynamic T1 High Resolution Isotropic Volume Excitation (THRIVE)—7 series every 33 s	4.1/1.97	24 × 24	120 × 120	6		1.5	Gd

**Table 2:** Main parameters of the Tofts model.

Quantity	Definition
*C*_*a*_(*t*)	Arterial concentration as a function of time
*C*_*t*_(*t*)	Tissue concentration as a function of time
*H*_*ct*_	Hematocrit volume
*K*_trans⁡_	Transfer constant from the blood plasma into the EES
*K*_*ep*_	Transfer constant from the EES back to the blood plasma
*T*	Onset time of arterial contrast uptake
*v*_*b*_	Whole blood volume per unit of tissue
*v*_*e*_	Total EES volume (*v*_*e*_ = *K*_trans⁡_/*K*_*ep*_)
